# Acute contact toxicity of insecticides for the chemical control of the invasive yellow-legged hornet *Vespa velutina nigrithorax* (Hymenoptera: Vespidae)

**DOI:** 10.1371/journal.pone.0320769

**Published:** 2025-04-16

**Authors:** Paula Malaquias Souto, Artur Sarmento, Nuno Capela, António Aguilar, Henrique M.V.S. Azevedo-Pereira, Cassiana Rebello Carvalho, Eric Darrouzet, Mélissa Haouzi, Luís António Henriques, Sara Leston, Fernando Ramos, José Paulo de Sousa

**Affiliations:** 1 Department of Life Sciences, Centre for Functional Ecology, Associated Laboratory TERRA, University of Coimbra, Coimbra, Portugal; 2 Município de Cantanhede, Praça Marquês de Marialva, Cantanhede, Portugal; 3 Centro Agroveterinário (CAV), Universidade do Estado de Santa Catarina (UDESC), Lages, Brazil; 4 Institut de Recherche sur la Biologie de l’Insecte, UMR CNRS, University of Tours, Parc de Grandmont Tours, 37200, France; 5 Associated Laboratory for Green Chemistry (LAQV), Network of Chemistry and Technology (REQUIMTE), Porto, Portugal; 6 Faculty of Pharmacy, University of Coimbra, Polo III, Coimbra, Portugal; University of Carthage, TUNISIA

## Abstract

The yellow-legged hornet, *Vespa velutina* subs. *nigrithorax* Buysson, 1905, originally from Southeast Asia, has become an invasive species in Europe since its introduction in France around 2004. Its rapid proliferation and voracious predatory behavior pose a significant threat to native insects, particularly honeybees and other pollinators, impacting agricultural production, biodiversity, and human safety. Eradication in Europe seems now impossible, and the control efforts are hindered by the lack of standardized application protocols, including for insecticide use, leading to potential indiscriminate pesticide application and, consequently, environmental damages. Our study evaluated the acute contact toxicity on *V. v. nigrithorax* workers of four commercially available formulations containing acetamiprid, cypermethrin, a mix of natural pyrethrins, and Spinosad as active ingredients. These tests were performed in laboratory conditions, offering novel data for the chemical control of this invasive species. Our results suggest acetamiprid and spinosad as promising candidates for the yellow-legged hornet control. Further research is needed to validate their efficacy under field conditions and assess ecological impacts of these pesticides on non-target organisms. Integrated pest management strategies should prioritize insecticides with low non-target toxicity and minimal environmental persistence to mitigate resistance development and ensure effective pest control. Comprehensive assessments considering multiple factors beyond mortality are essential for informing sustainable pest control strategies.

## Introduction

Exotic invasive species can imperil species, habitats, and even ecosystems, as many have the potential to adapt very quickly to new environments, which allows them to proliferate easily under new conditions [1]. Consequently, possible invasive species’ proliferation needs to be tackled, primarily, by defining and applying strategies to prevent their introduction. However, this is often difficult due to the unpredictability of these events. Once the invasion is identified, it is imperative to provide efforts to eradicate them as they can cause severe harm to the local ecosystems [2] and, ultimately, by having the potential to induce native species extinction [3]. However, when eradication is no longer feasible, managing the invasive species becomes necessary, with control strategies depending heavily on the local legal, economic, and social context, often leading to higher costs compared to early intervention efforts [4]. In Europe, it is estimated that invasive species had a total cost (direct and indirect) of €116.61 billion between 1960 and 2020 [5].

One of these exotic invasive species is the *Vespa velutina* subsp. *nigrithorax* Buysson, 1905 (Hymenoptera: Vespidae), commonly known as Asian hornet or the yellow-legged hornet. Originally from Southeast Asia, it is an invasive species in Europe, where was firstly introduced in France in 2004 [6]. The *V.v. nigrithorax* peculiar ecology is one of the reasons why it is very difficult to control this hornet species, as the primary nests (first queen posture) are small and not easily spotted, even in urban areas, and secondary nests (either fully developed or under development) are commonly settled in high tree’ canopies [7], even though they can also be found in many other places (including on the ground). Moreover, in areas where this species is considered invasive, there where records of adaptations of its life cycle, with active nests observed during winter and queens emerging from hibernation earlier than the period originally known for the species in its native range [8].

Eradication in Europe seems now impossible, with most countries focusing on controlling its spread through nest detection and destruction, particularly in urban and rural environments, and by protecting honeybee colonies [8]. Nest elimination is typically carried out in autumn because nests are more visible during this period than in summer, but by this stage, many new gynes may already be fertilized [7] and escape, potentially establishing new nests. The elimination process is also costly, with France spending an estimated €23M between 2006 and 2015 [9]. Effective control requires long-term management strategies, including early detection (prior to foundress fertilization) and proper methodologies for nest destruction [10,11].

Nest destruction, often using long-range equipment to inject insecticides (e.g., pyrethroids like permethrin), is the most common technique used in Portugal. Protein- and sugar-based baits are also used in poisoned bait systems (such as fipronil) [12]. Although these methods can be effective, they pose serious concerns, especially regarding the negative impact of pesticides on key ecosystem services like pollination and natural pest control, which are vital for maintaining biodiversity and ensuring food security [13]. The absence of species-specific protocols or authorized insecticides tailored to *V. v. nigrithorax* at the European level, has led to the use of pesticides and techniques that are either improvised or adapted from methods applied on other pest species, raising concerns about efficacy and environmental sustainability. Also, despite the intervention by the authorities, most nests that are destroyed, are not removed from the place due to the large costs involved in this procedure, thus becoming a potential environmental hazard if other species enter in contact with it or feed on dead adults/larvae [14], such as the European bee-eater (*Merops apiaster* Linnaeus, 1758) [15] and European honey buzzard (*Pernis apivorus* Linnaeus, 1758) [16–18].

In Portugal, the recommended chemical products for controlling *V. v. nigrithorax* nests are generally based on those used for broader wasp and hornet control, rather than being species-specific [19]. This approach means that these products are not tailored to effectively target *V. v. nigrithorax* and may not fully account for the ecological risks associated with their use on this invasive species. Furthermore, it is notable that no formal emergency authorizations for pesticides have been issued specifically for *V. v. nigrithorax* in the EU, as highlighted by the EU pesticides database [20]. This underscores a significant gap in regulation and available tools for addressing this invasive species. Therefore, it is of the utmost importance to proceed with new studies to (a) generate a methodological protocol for assessing the effects of pesticides on *V. v. nigrithorax* individuals and nests, and (b) test and select pesticides that effectively control this species while minimizing environmental harm.

In the present study, we tested four commercially available formulations under laboratory conditions for their acute contact toxicity on *V. v. nigrithorax* workers, which constitutes the major workforce of the colonies and that performs most tasks. The primary goal of pesticide nest treatments is to eliminate all the individuals, especially the queen, to prevent the colony from relocate [21]. However, using workers as test subjects is not a significant limitation, as their similar weight to queens [22] makes them a reliable proxy for evaluating pesticide effects, ensuring the applicability of the results to colony control strategies. The insecticides were selected according to three criteria: (i) active substance present in the list of biocides approved by the European Chemicals Agency (ECHA) [23]; (ii) active substance present in the list of authorized biocides by the *Direção-Geral de Saúde* of Portugal [24]; (iii) commercially available insecticides most commonly used to treat nests by authorities or certified companies in Portugal (e.g., Associação Nativa, Intermunicipal Communities of Coimbra region and of Viseu, Dão and Lafões). This study represents a tier 1 (laboratory) test, where each individual is exposed to the same amount of the product. The performed tests serve as an initial screening and dosage selection for future field applications, considering subsequent adjustments to better reflect real-world conditions.

## Materials and methods

### Ethics statement

Individuals of *V. v. nigrithorax* were collected from both private properties and public spaces. For collections on private properties, consent from the owners was obtained. In public areas, permission for collection was given by local authorities responsible for treating nests. Moreover, no special authorization is necessary to keep this species individuals in laboratory conditions, or to conduct insect animal testing within the EU. The fieldwork did not involve any endangered or protected species.

### Pesticide selection and characterization

The four insecticides selected were: Starpride Max^®^ (active ingredient – a.i. acetamiprid); Spintor^®^ (a.i. spinosad); Cythrin 10EC^®^ (a.i. Cypermethrin), and Pirecris^®^ (mix of natural pyrethrins as a.i.) (Table 1). The entire selection of the 4 formulations texted was also based on the high expected contact toxicity on hymenopterans (mainly bees - honeybees and bumblebees - which have more ecotoxicological data available), low toxicity for mammals and birds, and potential to stay in the market in the medium term. Information on relevant physical and chemical characteristics, namely persistence, was also considered (Table 1).

**Table 1:** Insecticides used in the study. Insecticide general information, physico-chemical characteristics and relevant ecotoxicity data (according to PPDB - Pesticide Properties DataBase [25] and BPDB - Bio-Pesticides DataBase [26]).

Commercial formulation	Starpride Max^®^	Spintor^®^	Cythrin 10EC^®^	Pirecris^®^
**Active ingredient (a.i.)**	Acetamiprid	Spinosad	Cypermethrin	Pyrethrin mix (cinerin I & II, jasmolin I & II and pyrethrin I & II)
**Concentration**	200 g a.i./L; 17.6% (w/w)	20 g a.i./L; 2.14% (w/w)	100 g a.i./L; 10.9% (w/w)	480 g a.i./L; 44% (w/w)
**Acute contact LD**_**50**_ **– Honeybee**	8.09 µg a.i./bee	0.0036 µg a.i./bee	0.023 μg a.i./bee	0.013 µg a.i./bee
**Acute oral LD**_**50**_ **– Mammals**	146 mg a.i./kg	>2000 mg a.i./kg	287 mg a.i./kg	700 mg a.i./kg
**Acute LD**_**50**_ **– Birds**	98 mg a.i./kg	>2000 mg a.i./kg	> 9520 mg a.i./kg	>51151 mg a.i./kg
**DT**_**50**_ **(lab at 20°C) – Soil**	1.6 days	15 days	117.7 days	2.5 days

LD_50_ – Lethal Dose for 50% of the population tested; DT_50_ – Half-life (time for an amount of a substance to be reduced by half through degradation).

### Adult acute contact toxicity tests

Adult acute contact toxicity tests with *V. v. nigrithorax* adult workers were performed according to a protocol adapted from the Organization for Economic Cooperation and Development – OECD [27,28], as described below.

#### Collection of hornets.

Adult workers were collected from active nests during October and November of 2023 (typically the secondary nest season goes from late Summer until Winter) in different districts of Portugal (S1 Table). In each nest, adults were collected using a sweep net in front of the nests (individuals were collected as soon as they exit the nest) and stored in 1,5L plastic bottles until further processing. In the laboratory, the bottles containing the hornets were placed in a freezer (−20ºC) to anesthetize them for safe handling. The time needed in the freezer varies depending on how long it takes for the hornets to have their metabolism reduced enough to be handled safely. Afterwards, the hornets were sexed and weighed to select females with 300–500 mg, which have a higher probability of being workers (the major component of the colony) [22].

Each pesticide was tested using five different nests (five replicates). For each test, six treatments were defined: five pesticide concentrations plus one control. For each treatment, 10 individuals were tested, totaling 60 per test and 300 per pesticide. For more information on the concentrations tested see the “Preparation of doses and exposure” section below.

#### Handling and feeding conditions.

Tests were conducted using 500 cm^3^ transparent plastic cages, perforated with several holes of approximately 2 mm to ensure sufficient ventilation for the survival of the individuals. Adult worker hornets were selected and individually placed in the cages (“single housing”) to prevent conflicts and undesired mortality, one day before the start of the test. They were kept under test conditions (detailed conditions below) inside an incubator until the next day for acclimation. Petri dishes (35 mm diameter) were used as feeders and placed inside the cages. Food consisted of a mixture of water, honey, and agar-agar in proportion of 1:0.25:0.0065, and was given *ad libitum* during the entire experiment.

Cages were kept inside an incubator equipped with a forced air-circulation system under constant dark and climatic conditions (suggested by the adopted protocols: 25 ± 2 °C and 60 ± 20% RH) throughout the complete duration of the test (except for unavoidable minor deviations, of maximum 2 hours, for mortality and behavioral assessment, and food replenishment). The tests had a duration of at least 48 hours after exposure (see below), being extended until 72 or 96 hours when controls’ mortality did not exceed 20% and the mortality of other treatments was still increasing [27–29].

#### Preparation of doses and exposure.

Five doses per test product were defined for testing based on the product toxicity data available. For each treatment – control and different concentrations of the test product – 10 hornets were tested. Stock and test solutions for each treatment were prepared with distilled water and 0.5% Triton X as surfactant [28]. Based on available toxicity data on other hymenopteran studies and preliminary data of our studies, the following concentrations were defined to help determine the lethal doses (LD_50_ and LD_90_): Starpride Max^®^: 0.025, 0.050, 0.100, 0.200, and 0.375 mg a.i/ml; Spintor^®^: 0.500, 0.900, 1.600, 2.800, and 5.000 mg a.i/ml; Cythrin 10EC^®^: 1.000, 1.500, 2.250, 3.350, and 5.000 mg a.i/ml; and Pirecris^®^: 0.750, 1.350, 2.350, 4.200, and 7.500 mg a.i./ml. Afterwards, the concentrations applied were quantified using different methods: mass spectrometry (GC-MS/MS) for Pirecris^®^ (mix of natural pyrethrins) samples; High-Performance Liquid Chromatography-Ultraviolet (HPLC-UV) for Starpride MAX^®^ (acetamiprid) and Spintor^®^ (spinosad) samples; and Ultra Performance Liquid Chromatography (UPLC-MS) for Cythrin 10EC^®^ (cypermethrin) samples. Ultimately, based on those analysis we came to the conclusion that the concentrations applied (“real concentrations”) were: Starpride Max^®^: 0.023, 0.046, 0.092, 0.184, and 0.345 mg a.i/ml; Spintor^®^: 0.396, 0.713, 1.267, 2.217, and 3.959 mg a.i/ml; Cythrin 10EC^®^: 0.917, 1.376, 2.063, 3.072, and 4.585 mg a.i/ml; and Pirecris^®^: 0.420, 0.756, 1.316, 2.352, and 4.200 mg a.i./ml. Control individuals were treated with a pesticide-free solution containing distilled water and 0.5% Triton X.

During pesticide application, each hornet was anaesthetized using cold as described above (for a second time) and treated by topical application. A droplet of 2 μl containing the test solution was applied to the dorsal side of the thorax using a Hamilton syringe equipped with a repeating dispenser. Afterwards, the cages were placed again in the incubator. Mortality and abnormal behavior (see below) were recorded after exposure (4–6h) and then every 24h (24, 48 and 72, and 96h, if applicable). Each experiment was terminated by freezing the test cages (with individuals inside) at ≤ -20°C.

#### Abnormal behaviors.

To quantify the number of hornets exhibiting abnormal behavior per cage per day, we established a nomenclature based on standard ecotoxicological guidelines (e.g., [29]), and on observer notes from a preliminary test. Each cage was observed for approximately 30 seconds and the behavior registered according to the following categories: Apathy (hornets show only low or delayed reactions to stimulation with long periods of immobility); Hyperactivity (when compared to controls); Affected (hornets show signs of reduced coordination; contract abdomen or entire body; sometimes affected individuals can recover); Moribund (hornets cannot walk, often backwards and show only very feeble movements of legs and antennae; usually leads to the death of the individual).

### Statistical analysis

To determine the lethal doses to eliminate 50% (LD_50_) and 90% (LD_90_) of the population tested (mg a.i./ml and μg a.i./hornet), results were statistically analyzed, according to OECD [27–29], by probit analysis using PriProbit software version 1.63 [30]. We applied Abbott’s correction for control mortality rates when there was natural or random death in controls [31]. Means and standard error of mortality were calculated using the R program [32] and the “ggplot2” package [33] was used to visualize the data, by generating the plots presented in the Results section.

## Results

### Mortality and lethal doses

**Fig 1:**
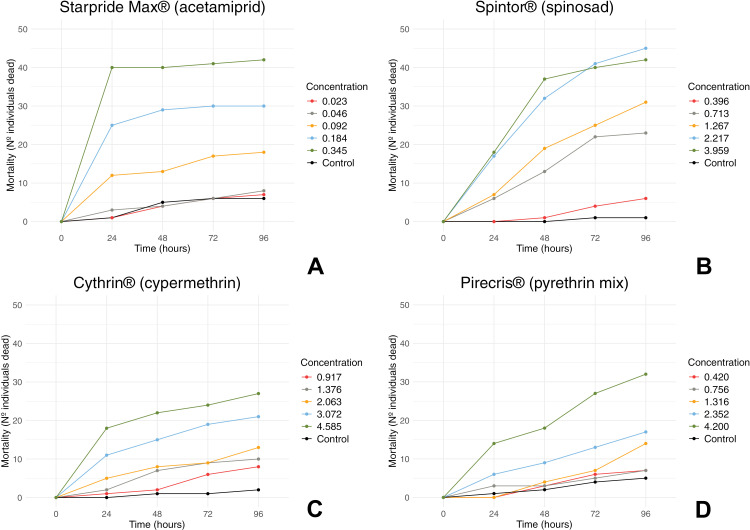
Mortality (Nº individuals dead) of *Vespa velutina nigrithorax* topically exposed to 2μl of four different pesticides. A–D, Mortality curves for acetamiprid (A), spinosad (B), cypermethrin (C), and mix of natural pyrethrins (D). Concentrations are in mg a.i/ml.

The acute lethal toxicity results revealed distinct toxicity patterns toxicity levels (LD_50_ and LD_90_ values) among the tested pesticides against *V. v. nigrithorax* (Table 2). All pesticides achieved the highest mortality at the highest concentration tested, except for Spintor^®^ (a.i. spinosad, Fig 1, S2–S5 Tables).

**Table 2:** Acute lethal toxicity (LD_50_ and LD_90_) of formulations topically applied to the yellow-legged hornet *Vespa velutina nigrithorax.* Observations were made in four time points: 24, 48, 72, and 96 hours post-exposure to the insecticide. LD values are presented in two units: mg a.i./ml and μg a.i./hornet. The values in parentheses indicate the 95% confidence interval values for LD.

Insecticide	LD50	LD90	LD50	LD90
	(mg a.i./ml)	(mg a.i./ml)	(μg a.i./hornet)	(μg a.i./hornet)
**ST**				
24h	0.206	0.454	0.411	0.905
	(0.139, 0.353)	(0.2885, 2.131)	(0.277, 0.704)	(0.576, 4.251)
48h	0.187	0.449	0.373	0.896
	(0.165, 0.213)	(0.368, 0.595)	(0.330, 0.425)	(0.734, 1.188)
72h	0.175	0.438	0.349	0.873
	(0.154, 0.200)	(0.357, 0.582)	(0.308, 0.399)	(0.712, 1.161)
96h	0.162	0.488	0.324	0.973
	(0.141, 0.189)	(0.383, 0.682)	(0.735, 1.474)	(0.735, 1.474)
**SP**				
24h	5.290	32.496	10.581	65.036
	(3.784, 9.480)	(15.626, 130,226)	(7.197, 22.533)	(28.199, 398.729)
48h	1.738	6.426	3.475	12.856
	(1.496, 2.050)	(4.816, 9.781)	(2.907, 4.249)	(9.204, 21.725)
72h	1.196	4.617	2.391	9.235
	(0.699, 1.942)	(2.581, 27.535)	(0.9138, 5.360)	(4.500, 687.696)
96h	0.977	3.477	1.953	6.955
	(0.547, 1.503)	(2.075, 14.928)	(0.662, 3.769)	(3.650, 192.059)
**CY**				
24h	6.305	19.909	12.611	39.825
	(4.939, 9.750)	(12,066, 51.988)	(9.526, 22.226)	(22.497, 139.896)
48h	5.465	19.724	10.931	39.461
	(4.343, 8.097)	(11.905, 50.633)	(8.390, 18.140)	(22.180, 134.388)
72h	5.256	27.384	10.513	54.781
	(4.023, 8.574)	(14.228, 103.288)	(7.731, 19.969)	(26.013, 317.887)
96h	4.482	22.633	8.963	45.273
	(3.552, 6.670)	(12.531, 71.868)	(6.853, 15.013)	(23.083, 206.685)
**PI**				
24h	8.982	36.508	17.964	73.016
	(5.953, 21.261)	(16.818, 202.833)	(11.242, 57.225)	(30.334, 741.715)
48h	7.637	34.402	15.261	68.804
	(5.278, 15.195)	(16.744, 144.177)	(10.995, 37.654)	(30.311, 455.690)
72h	5.025	19.931	10.049	39.862
	(3.050, 27.371)	(7.765, 1,144.490)	(5.357, 2,939.760)	(12.846, 5.526e+7)
96h	3.677	14.886	7.354	29.773
	(2.984, 4.944)	(9.597, 30.215)	(5.765, 10.653)	(17.964, 73.065)

a.i.: active ingredient. ST: Starpride MAX^®^ (a.i. acetamiprid); SP: Spintor^®^ (a.i. spinosad); CY: Cythrin 10EC^®^ (a.i. cypermethrin); PI: Pirecris^®^ (a.i. mix of natural pyrethrins).

Starpride Max^®^ (a.i. acetamiprid) displayed the highest toxicity, presenting the lowest LD_50_ and LD_90_ values among the pesticides tested (ST, Table 2). Acetamiprid demonstrated increasing mortality with higher concentrations, with the highest mortality observed at 0.345 mg a.i./ml (highest concentration) after 96 hours of exposure (Fig 1A). In fact, throughout the bioassay, Starpride Max^®^ showed high toxicity, especially when compared with the other commercial formulations tested. Although the highest mortality (84%, 42/50) was achieved after 96 hours of topical application, after 24 hours it was already possible to observe a mortality rate of 80% (40/50) of the individuals tested (Fig 1A).

Spintor^®^ (a.i. spinosad) was the second most toxic pesticide based on LD values (SP, Table 2). The mortality rate increased with both concentration and time, reaching highest levels at the two highest concentrations: 2.217 mg a.i./ml (90%, 45/50) and 3.959 mg a.i./ml (84%, 42/50) (Fig 1B). Spinosad also showed notable toxicity to control *V. v. nigrithorax*, killing more individuals in total (90%, 45/50) than the most toxic compound tested, acetamiprid (84%, 42/50). However, its action is slower comparing to Starpride Max^®^ (i.a. acetamiprid), taking more than 24 hours to eliminate >50% of the hornets tested (Fig 1B).

Cythrin 10EC^®^ (a.i. cypermethrin) exhibited low toxicity compared to other pesticides (CI, Table 2), with the lowest mortality rate among all the tested products. Mortality increased with concentration and time, reaching its maximum of 54% (26/50) at 4.585 mg a.i./ml after 96 hours (Fig 1C). Similarly, Pirecris^®^ (a.i mixture of natural pyrethrins) displayed low toxicity (PI, Table 2). As observed for spinosad (Fig 1B) and cypermethrin (Fig 1C), the action of the pyrethrin mix was slow, gradually increasing mortality over time (Fig 1D).

### Abnormal behaviors

The predominant abnormal behavior detected during the bioassay for all pesticides were “affected” and “moribund”. “Locomotor difficulties” were exclusively noted in individuals exposed to the active compounds cypermethrin and the mix of natural pyrethrins. “Cramps” were observed sporadically in individuals subjected to cypermethrin bioassay. Occasions of “apathy” were limited to bioassays involving spinosad and the mix of natural pyrethrins. In contrast, “hyperactivity” was exclusively observed in individuals exposed to cypermethrin and spinosad.

## Discussion

The present study provides the first evaluation of the effectiveness of insecticides in controlling *Vespa velutina nigrithorax* based on laboratory ecotoxicological tests. Our results suggest the products containing acetamiprid and spinosad are promising candidates for the control of the yellow-legged hornet. However, as a Tier 1 screening study, our research requires further validation under field-like conditions. The tests performed were focused on individual hornets rather than entire nests, highlighting the need for additional studies to evaluate the effectiveness of the above-mentioned molecules in more realistic scenarios and their impact on non-target organisms.

On the other hand, by testing commercially available products rather than isolated active ingredients, we aimed to simulate real-world conditions, where insecticides are typically applied as formulations. The effects of insecticides on non-target species can vary significantly when tested as formulations, due to the potential influence of co-formulants on the toxicity profile of the active ingredient [34–36]. Testing commercial formulations also provides a more comprehensive understanding of the risks posed to non-target organisms under field conditions [36].

In real-world pesticide application, such as nest treatments, the LD_90_ values obtained in this study should be interpreted with caution due to the high variability in confidence intervals – except for Starpride Max^®^ (a.i. acetamiprid). While LD_50_ is crucial for comparing toxicity between and across organisms and species, LD_90_ was included to approximate the dose required to inactivate a nest, as effective control requires eliminating around 90% of the adults.

Kishi & Goka [37] reviewed case studies on chemical control of invasive vespine wasps (Hymenoptera, Vespidae, Vespinae) and discussed potential techniques and insecticides for controlling *V. v. nigrithorax* in Japan. Among the insecticides evaluated were two of the active compounds tested in the present study, acetamiprid and spinosad, which, according to our experiments, proved promising in the chemical control of *V. v. nigrithorax*. Nonetheless, in the mentioned study, these two insecticides were considered ineffective and highly repellent when associated with food baits – oral exposure [38]. Since our experiments were based on individuals contact exposure, it was not possible to test whether they are in fact repellent or not. Therefore, ecotoxicological studies with oral exposure, in addition to validation of laboratory data in the field, are needed to assess the repellency or effectiveness, when applied via direct injection/spray of the formulation into the nests comparing to when applied orally via food baits.

Considering that insecticide injection is a common method for destroying *V. v. nigrithorax* nests, a substance with long-lasting residual activity is desirable, as it ensures continued effectiveness even when not all hornets are present in the nest during treatment. However, this prolonged activity increases the risk of exposure for non-target organisms, particularly predators and scavengers. For instance, European bee-eaters (*Merops apiaster*) and European honey buzzard (*Pernis apivorus*), which have been reported as natural predators of this invasive species [15–18], may be exposed to the substance if they consume contaminated hornets. Despite the lack of information regarding the presence and the effects of pesticide substances in these predator species, there is already some evidence of neonicotinoid residues in the European honey buzzard (8 out of 10 blood samples tested were positive) [39]. In this context, broader ecological implications, particularly in the context of its persistence in the environment and its potential impact on non-target species, must be carefully considered.

Our results demonstrated that Starpride Max^®^ (a.i. acetamiprid), even at low dosages, was highly effective, killing a significant portion of the tested population within the first 24 hours. Neonicotinoid-based pesticides (e.g., acetamiprid, thiamethoxam, imidacloprid, thiacloprid, and clothianidin) have become the most widely used insecticides worldwide in recent years [40]. However, in the European Union, their use has been largely restricted mainly due to high toxicity to pollinators, especially the honeybee *Apis mellifera* [41,42]. Despite these restrictions, many neonicotinoids have continued to be used under emergency authorization processes, raising concerns about their environmental impact and effects on non-target species [13]. Neonicotinoids target insect nicotinic acetylcholine receptors, causing neural hyperexcitation, paralysis, and eventually death [43], behaviors observed in our study. Acetamiprid is often regarded as a safer alternative among neonicotinoids due to its very low to moderate persistence in soil [44] and its low toxicity to honeybees [45,46]. However, despite being specifically designed to target insects, its extensive use has raised concerns about risks to mammals and other non-target organisms, including humans [47]. First-tier risk assessments indicate low acute risk for mammals and some bird species, but they also reveal high long-term risks for small herbivorous mammals and insectivorous birds [48]. Furthermore, research suggests that acetamiprid can cause reproductive toxicity in both male and female mammals, as well as genotoxic, neurotoxic, and reprotoxic effects [47]. Documented cases of acute acetamiprid poisoning in humans [49–51] underscore the importance of using appropriate protective equipment as well as proper pesticide containment during activities such as nest control, as inadequate handling may increase health risks.

Similarly, Spintor^®^ (a.i. spinosad), showed potential for the chemical control of *V. v. nigrithorax* based on our findings. Its mode of action is via the activation of nicotinic acetylcholine receptors and GABA (gamma-aminobutyric acid) receptors in the central and peripheral nervous systems of insects [52]. This activation causes neural hyperexcitation, leading to paralysis and death of the target insects, behaviors observed in our study. It is derived from the fermentation of the soil bacterium *Saccharopolyspora spinosa*, consisting of a complex mixture of active compounds, mainly spinosad A and spinosad D [53]. With growing interest in biological pesticides for integrated pest management, spinosad has gained attention as a potential sustainable alternative to reduce the use of persistent pesticides [54,55]. Often classified as a compound with reduced environmental and toxicological risk [56], it exhibits low toxicity to birds and mammals [57,58], which are non-target groups that may come into contact with pesticide-treated nests and hornets. However, studies have shown that spinosad can harm beneficial arthropods (e.g., parasitoids, predators, pollinators), causing high mortality, poisoning symptoms, and reduced mobility. These effects vary depending on concentration, exposure method, and the species affected [59]. Laboratory studies indicate high toxicity to honeybees even at low concentrations [60–63], suggesting adverse effects on energy production and metabolism [63], in addition to being toxic to vital organs, impairing locomotion [62], which negatively impacts worker foraging. Nevertheless, it is possible that under more realistic conditions of application of this pesticide in the field, the toxicity of foraging bees may be considered insignificant once the residues have completely dried [60,64].

Although low toxicity was found for Cythrin 10EC^®^ (a.i. cypermethrin) and Pirecris^®^ (a.i. mix of natural pyrethrin), both insectitides caused negative effect on locomotion, which may suggest negative consequences for the essential activities of *V. v. nigrithorax* workers. These insecticides act by interacting with sodium channels in insect nerve membranes, leading to neural hyperexcitation, paralysis, and eventual death [65,66]. Cypermethrin is a pyrethroid insecticide, a synthetic derivative of natural pyrethrins found in certain plants, developed to enhance photostability and preserve the insecticidal potency and low mammalian toxicity of the natural compounds, which degrade quickly under light exposure [66,67]. While both pyrethrins and cypermethrin are considered low-risk to birds, mammals [68,69], and generally safe to human health, prolonged exposure through skin contact, inhalation, or open wounds increases toxicity to mammals [70]. The risk of acute illness has risen with their widespread agricultural use, particularly due to spills, improper storage, and inadequate evacuation during pesticide application [71]. In this context, workers handling *V. v. nigrithorax* nest deactivation are particularly vulnerable. Chronic effects and high toxicity to beneficial insects, especially pollinators, have been widely reported [36] and must be also considered. Even if pollinators do not directly contact treated nests, pesticide spillage could still occur, and the long environmental persistence of cypermethrin may exacerbate the risk, causing lasting ecological consequences for non-target species.

In summary, an approach that considers the risks and benefits of each pesticide is essential for effective pest management and environmental protection, and the control *V. v. nigrithorax* is no exception. We recommend that the choice of insecticides to be used in the control of *V. v. nigrithorax* should consider the following factors: (i) toxicity to non-target organisms, especially to pollinators and potential predators; (ii) low persistence in the environment; (iii) alternation of the compound used to avoid resistance. Thus, based on the present study, we currently suggest, among the five possibilities tested, the use of the insecticides acetamiprid and spinosad. Nonetheless, their use should be done with caution, as ecotoxicological studies using oral exposure with adults and larvae are still needed, as well as contact exposure with larvae, in addition to semi-field and field studies that present a more realistic scenario with the application of the insecticides directly into the nest.

## Conclusions

In conclusion, this study offers a comprehensive evaluation of insecticide effectiveness in controlling *Vespa velutina nigrithorax* based on ecotoxicological laboratory tests. While our results suggest acetamiprid and spinosad as promising options for nest control, it is important to note the limitations of our approach, including the use of individual hornets rather than entire nests and the testing of commercially available formulations instead of isolated active ingredients. Also, lethal dose values should be interpreted with caution due to variability in confidence intervals. Their use in real-world applications, such as nest treatments, must account for these uncertainties. These factors highlight the need for further research to validate the efficacy of these compounds in more complex, field-like conditions and to assess their impact on both target and non-target species. We believe that these results will contribute to the development of integrated pest management strategies for this species in regions where it is considered invasive and poses environmental, economic, and public health threats. The findings underscore the importance of considering factors beyond mortality when assessing pesticide efficacy, such as toxicity to non-target organisms and environmental persistence.

## Supporting information

S1 Table Description of the sites where the *Vespa velutina nigrithorax* nests for adult collection were located. *Depending on the nest size (i.e., number of workers available for testing), it was possible to test more than one pesticide with hornets from the same nest.
(DOCX)

S2 Table Mortality data (number of dead individuals) of *Vespa velutina nigrithorax* in the 5 nests collected for each concentration of Starpride MAX^®^ over the time observed during the test. These values were used for calculations in Priprobit and to generate mortality curves. a.i.: active ingredient.
(DOCX)

S3 Table Mortality data (Nº individuals dead) of *Vespa velutina nigrithorax* in the 5 nests collected for each concentration of Spintor^®^ over the time observed during the test. These values were used for calculations in Priprobit and to generate mortality curves. a.i.: active ingredient.
(DOCX)

S4 Table Mortality data (Nº individuals dead) of *Vespa velutina nigrithorax* in the 5 nests collected for each concentration of Cythrin^®^ over the time observed during the test. These values were used for calculations in Priprobit and to generate mortality curves. a.i.: active ingredient.
(DOCX)

S5 Table Mortality data (Nº individuals dead) of *Vespa velutina nigrithorax* in the 5 nests collected for each concentration of Pirecris^®^ over the time observed during the test. These values were used for calculations in Priprobit and to generate mortality curves. a.i.: active ingredient.
(DOCX)
