# Supplementary material for: Acute contact toxicity of insecticides for the chemical control of the invasive yellow-legged hornet Vespa velutina nigrithorax (Hymenoptera: Vespidae)
Source: PLoS One. 2025 Apr 16;20(4):e0320769. doi: 10.1371/journal.pone.0320769 (PMC12002430; doi:10.1371/journal.pone.0320769)
Supplement: S1 Table — *Depending on the nest size (i.e., number of workers available for testing), it was possible to test more than one pesticide with hornets from the same nest. (DOCX) [file pone.0320769.s001.docx]

**S1 Table. Description of the sites where the *Vespa velutina nigrithorax* nests for adult collection were located.** *****Depending on the nest size (i.e., number of workers available for testing), it was possible to test more than one pesticide with hornets from the same nest.

| **Date** | **Location** | **Geographical coordinates** | **Formulation tested*** |
| --- | --- | --- | --- |
| 04/X/2023 | Portugal, Coimbra | 40º12'40.8"N 8º27'53.1"W | Spintor, Pirecris |
| 06/X/2023 | Portugal, Coimbra | 40º11'24.4"N 8º24'5.5"W | Spintor, Pirecris |
| 10/X/2023 | Portugal, Aveiro, Águeda | 40º39'48.9"N 8º27'25.3"W | Spintor, Pirecris |
| 12/X/2023 | Portugal, Aveiro, Águeda | 40°34'14.0"N 8°25'50.5"W | Cythrin |
| 16/X/2023 | Portugal, Coimbra | 40°15'48.0"N 8°27'40.9"W | Cythrin |
| 20/X/2023 | Portugal, Góis | 40°08'51.1"N 8°08'17.1"W | Pirecris |
| 27/X/2023 | Portugal, Lousã | 40°07'01.1"N 8°13'46.8"W | Cythrin, Starpride MAX, Pirecris, Spintor |
| 31/X/2023 | Portugal, Aveiro, Águeda | 40°35'47.7"N 8°29'51.8"W | Cythrin, Spintor |
| 31/X/2023 | Portugal, Aveiro, Águeda | 40°35'34.8"N 8°29'43.1"W | Starpride MAX |
| 02/XI/2023 | Portugal, Aveiro, Águeda | 40º32'6.828"N 8º27'53.1"W | Cythrin, Starpride MAX |
| 06/XI/2023 | Portugal, Aveiro, Águeda | 40°32'53.7"N 8°26'59.8"W | Starpride MAX |
| 09/XI/2023 | Portugal, Coimbra | 40°07'24.0"N 8°28'37.1"W | Starpride MAX |
