# Supplementary material for: Acute contact toxicity of insecticides for the chemical control of the invasive yellow-legged hornet Vespa velutina nigrithorax (Hymenoptera: Vespidae)
Source: PLoS One. 2025 Apr 16;20(4):e0320769. doi: 10.1371/journal.pone.0320769 (PMC12002430; doi:10.1371/journal.pone.0320769)
Supplement: S3 Table — These values were used for calculations in Priprobit and to generate mortality curves. a.i.: active ingredient. (DOCX) [file pone.0320769.s003.docx]

**S3 Table. Mortality data (Nº individuals dead) of *Vespa velutina* *nigrithorax* in the 5 nests collected for each concentration of Spintor® over the time observed during the test.** These values ​​were used for calculations in Priprobit and to generate mortality curves. a.i.: active ingredient.

|  |  |  | **Nº individuals dead** | | | | |
| --- | --- | --- | --- | --- | --- | --- | --- |
| **Nest** | **Treatment** | **Concentration mg a.i/ml** | **24h** | **48h** | **72h** | **96h** | **Total** |
| 1 | C1 | 0.396 | 0 | 0 | 0 | 0 | 0 |
| 1 | C2 | 0.713 | 1 | 1 | 3 | 1 | 6 |
| 1 | C3 | 1.267 | 1 | 4 | 2 | 0 | 7 |
| 1 | C4 | 2.217 | 3 | 2 | 4 | 0 | 9 |
| 1 | C5 | 3.959 | 2 | 5 | 0 | 0 | 7 |
| 2 | Control | 0.000 | 0 | 0 | 0 | 0 | 0 |
| 2 | C1 | 0.396 | 0 | 0 | 1 | 1 | 2 |
| 2 | C2 | 0.713 | 1 | 1 | 1 | 0 | 3 |
| 2 | C3 | 1.267 | 2 | 0 | 0 | 3 | 5 |
| 2 | C4 | 2.217 | 4 | 1 | 2 | 1 | 8 |
| 2 | C5 | 3.959 | 0 | 1 | 2 | 2 | 5 |
| 3 | Control | 0.000 | 0 | 0 | 0 | 0 | 0 |
| 3 | C1 | 0.396 | 0 | 1 | 1 | 1 | 3 |
| 3 | C2 | 0.713 | 3 | 2 | 2 | 0 | 7 |
| 3 | C3 | 1.267 | 3 | 4 | 1 | 0 | 8 |
| 3 | C4 | 2.217 | 5 | 4 | 0 | 0 | 9 |
| 3 | C5 | 3.959 | 6 | 4 | 0 | 0 | 10 |
| 4 | Control | 0.000 | 0 | 0 | 0 | 0 | 0 |
| 4 | C1 | 0.396 | 0 | 0 | 0 | 0 | 0 |
| 4 | C2 | 0.713 | 0 | 0 | 1 | 0 | 1 |
| 4 | C3 | 1.267 | 0 | 2 | 2 | 2 | 6 |
| 4 | C4 | 2.217 | 0 | 4 | 3 | 2 | 9 |
| 4 | C5 | 3.959 | 3 | 6 | 1 | 0 | 10 |
| 5 | Control | 0.000 | 0 | 0 | 1 | 0 | 1 |
| 5 | C1 | 0.396 | 0 | 0 | 1 | 0 | 1 |
| 5 | C2 | 0.713 | 1 | 3 | 2 | 0 | 6 |
| 5 | C3 | 1.267 | 1 | 2 | 1 | 1 | 5 |
| 5 | C4 | 2.217 | 5 | 4 | 0 | 1 | 10 |
| 5 | C5 | 3.959 | 7 | 3 | 0 | 0 | 10 |
