# Supplementary material for: Acute contact toxicity of insecticides for the chemical control of the invasive yellow-legged hornet Vespa velutina nigrithorax (Hymenoptera: Vespidae)
Source: PLoS One. 2025 Apr 16;20(4):e0320769. doi: 10.1371/journal.pone.0320769 (PMC12002430; doi:10.1371/journal.pone.0320769)
Supplement: S4 Table — These values were used for calculations in Priprobit and to generate mortality curves. a.i.: active ingredient. (DOCX) [file pone.0320769.s004.docx]

**S4 Table. Mortality data (Nº individuals dead) of *Vespa velutina* *nigrithorax* in the 5 nests collected for each concentration of Cythrin® over the time observed during the test.** These values ​​were used for calculations in Priprobit and to generate mortality curves. a.i.: active ingredient.

|  |  |  | **Nº individuals dead** | | | | |
| --- | --- | --- | --- | --- | --- | --- | --- |
| **Nest** | **Treatment** | **Concentration mg a.i/ml** | **24h** | **48h** | **72h** | **96h** | **Total** |
| 1 | C1 | 0.917 | 0 | 0 | 2 | 0 | 2 |
| 1 | C2 | 1.376 | 0 | 2 | 0 | 0 | 2 |
| 1 | C3 | 2.063 | 0 | 0 | 0 | 2 | 2 |
| 1 | C4 | 3.072 | 2 | 0 | 0 | 2 | 4 |
| 1 | C5 | 4.585 | 5 | 1 | 0 | 0 | 6 |
| 2 | Control | 0 | 0 | 0 | 0 | 0 | 0 |
| 2 | C1 | 0.917 | 0 | 0 | 0 | 1 | 1 |
| 2 | C2 | 1.376 | 0 | 0 | 0 | 0 | 0 |
| 2 | C3 | 2.063 | 1 | 0 | 0 | 0 | 1 |
| 2 | C4 | 3.072 | 1 | 2 | 1 | 0 | 4 |
| 2 | C5 | 4.585 | 3 | 1 | 0 | 0 | 4 |
| 3 | Control | 0 | 1 | 1 | 1 | 1 | 4 |
| 3 | C1 | 0.917 | 1 | 1 | 1 | 1 | 4 |
| 3 | C2 | 1.376 | 1 | 2 | 1 | 1 | 5 |
| 3 | C3 | 2.063 | 2 | 3 | 1 | 1 | 7 |
| 3 | C4 | 3.072 | 6 | 1 | 1 | 1 | 9 |
| 3 | C5 | 4.585 | 4 | 1 | 2 | 1 | 8 |
| 4 | Control | 0 | 0 | 0 | 0 | 0 | 0 |
| 4 | C1 | 0.917 | 0 | 0 | 0 | 0 | 0 |
| 4 | C2 | 1.376 | 0 | 0 | 0 | 0 | 0 |
| 4 | C3 | 2.063 | 0 | 0 | 0 | 0 | 0 |
| 4 | C4 | 3.072 | 2 | 1 | 1 | 0 | 4 |
| 4 | C5 | 4.585 | 3 | 0 | 0 | 2 | 5 |
| 5 | Control | 0 | 0 | 1 | 0 | 0 | 1 |
| 5 | C1 | 0.917 | 1 | 0 | 1 | 0 | 2 |
| 5 | C2 | 1.376 | 1 | 1 | 2 | 0 | 4 |
| 5 | C3 | 2.063 | 2 | 0 | 0 | 1 | 3 |
| 5 | C4 | 3.072 | 0 | 1 | 1 | 0 | 2 |
| 5 | C5 | 4.585 | 3 | 2 | 0 | 1 | 6 |
